# Developmental and lifelong exposure to mercury chloride exacerbates cognitive and motor decline in aged mice

**DOI:** 10.3934/Neuroscience.2026007

**Published:** 2026-03-10

**Authors:** Hafsa Malqui, Meriem Laaroussi, Hammou Anarghou, Oumaima Essaidi, Laila Berroug, Mounir Cherkaoui, El Hachmi Er-rachdaoui, Latifa Talhaoui, Fatiha Chigr

**Affiliations:** 1 Biological Engineering Laboratory, Faculty of Science and Technology, Sultan Moulay Slimane University, Beni Mellal, Morocco; 2 Polydisciplinary Faculty of Khouribga, Sultan Moulay Sliman University, Beni Mellal, Morocco; 3 High Institute of Nursing Professions and Health Techniques, Guelmim, Morocco

**Keywords:** mercury chloride, chronic exposure, memory, aging

## Abstract

Aging is a multifactorial biological process characterized by the progressive decline of cellular, tissue, and organ functions, leading to increased vulnerability to neurodegenerative disorders. Among the proposed mechanisms, the oxidative stress theory of aging remains one of the most widely accepted, emphasizing the role of reactive oxygen species (ROS) accumulation, particularly in the central nervous system (CNS). Mercury is a persistent environmental pollutant known to induce oxidative damage, but its contribution to age-related neuronal decline remains poorly understood. Our aim of this study was to investigate whether chronic HgCl_2_ exposure exacerbates age-related decline in memory and locomotor functions in aged male mice. To this end, mice were divided into two groups: A control group, which received only tap water, and an HgCl_2_-exposed group, which received 40 ppm HgCl_2_ in their drinking water daily from gestation until 18 months of age. At the end of the exposure, mice were subjected to behavioral tests to assess memory and locomotor activity. Following these behavioral assessments, oxidative stress parameters and acetylcholinesterase (AChE) activity were measured in the hippocampus and cerebellum. Our results showed that HgCl_2_ impaired locomotor activity in the Open Field test (OF) and memory performance in the Y-maze and Novel Object Recognition (NOR) test. These deficits were accompanied by a significant increase in malondialdehyde (MDA) and acetylcholinesterase (AChE) activity in the hippocampus. Furthermore, mercury exposure led to an increase in MDA and a significant upregulation of catalase (CAT) and superoxide dismutase (SOD) activities in the cerebellum. These changes were also associated with a marked decrease in AChE activity within the same region. Overall, these findings suggest that lifelong mercury exposure accelerates neurobehavioral decline by exacerbating oxidative and cholinergic disturbances, providing insight into how early and sustained exposure to environmental toxicants may promote premature brain aging.

## 1. Introduction

Aging is an irreversible biological process characterized by the progressive decline in cellular, tissue, and organ functions, which promotes the development of diseases. It is governed by intrinsic factors, such as genetic and molecular mechanisms, and extrinsic factors, including environmental influences and lifestyle choices [1]. This process is accompanied by the accumulation of damage to lipids, proteins, and nucleic acids [2]–[4]. Generally, aging is associated with brain atrophy, alterations in functional brain responses [5]–[8], and cognitive decline [9],[10].

The oxidative stress, or free radical, theory of aging, initially proposed by D. Harman [11],[12], is one of the most widely accepted explanations for the mechanisms of aging. The brain is particularly susceptible to oxidative damage because it consumes approximately 20% of the body's total oxygen and contains post-mitotic neurons that cannot regenerate after injury, leading to progressive mitochondrial dysfunction over time [13],[14]. This vulnerability results in the accumulation of oxidative damage markers, including DNA lesions, impaired protein metabolism, lipid peroxidation, and toxic metals such as mercury [15]–[17]. Reactive oxygen species (ROS) may be endogenous, arising from cellular metabolism, or exogenous, originating from sources such as radiation, infections, pesticides, environmental toxins, UV exposure, tobacco smoke [18], and heavy metals, particularly mercury [17].

Mercury, a commonly used heavy metal, is well known for its significant toxicity, the severity of which is directly correlated with the level of exposure. This metal exists in multiple forms: Metallic mercury (vapor), organic compounds such as methylmercury (MeHg), and inorganic salts like HgCl₂, each exhibiting a distinct toxicity profile [19]. Even at low doses, chronic exposure can lead to progressive accumulation and subclinical effects across organ systems [20]. Mercury is strongly suspected of promoting oxidative stress in the brain, potentially contributing to neurological disorders and accelerating the aging process, especially given its widespread presence in the environment particularly in food (notably fish), dental amalgams, and certain cosmetics [21].

Regarding inorganic mercury, several researchers have assessed its impact on the CNS and reported behavioral and cognitive impairments, including memory deficits, anxiety, depression, and impaired social interactions [22],[23]. In our previous study [24], we also demonstrated the neurotoxicity of this form of mercury following exposure beginning during the gestational period and continuing into adulthood.

The cerebral cholinergic system, composed of neurons using acetylcholine as a neurotransmitter, is primarily in the basal forebrain, striatum, and brainstem, with widespread projections to the cortex, hippocampus, and motor-related structures [25]. It plays a key role in hippocampal network modulation, regulating neuronal excitability, synaptic plasticity, and network oscillations via muscarinic and nicotinic receptors, which are essential for attention, learning, and memory [26]. In the cerebellum, acetylcholine modulates neuronal circuits and deep cerebellar nuclei, contributing to motor coordination and motor learning, as shown by studies where cholinergic inhibition in the interposed nucleus impairs motor performance [27],[28]. Aging is associated with a gradual decline in cholinergic transmission and neuronal loss, contributing to cognitive and motor deficits [29]. Furthermore, exposure to inorganic mercury (HgCl_2_) can inhibit AChE by binding to thiol groups, disrupting acetylcholine degradation and central cholinergic transmission, leading to cognitive motor impairments observed in experimental models [30].

In this study, we extend our prior study conducted in adult mice (3 months old) [24], and aim to investigate the effects of prolonged low-dose mercuric chloride exposure beginning in utero and continuing into advanced age (18 months). Our objective is to determine whether such chronic exposure affects cognitive functions known to be vulnerable to aging, particularly memory and locomotor activity, and to identify the underlying biochemical mechanisms associated with these alterations.

## 2. Materials and methods

### 2.1. Animal housing, treatment, and study design

Adult female and male Swiss albino mice were obtained from the animal facility of the Faculty of Sciences and Technology, Beni Mellal, Morocco. Animals were housed in standard plastic cages (30 cm × 15 cm × 12 cm) and kept under constant room temperature (22 ± 1 °C), humidity (55% ± 5%), and ventilation. Furthermore, mice were maintained in a 12-h light/dark cycle (lights on 8 a.m. to 8 p.m.) with free access to food and water.

**Figure 1.**
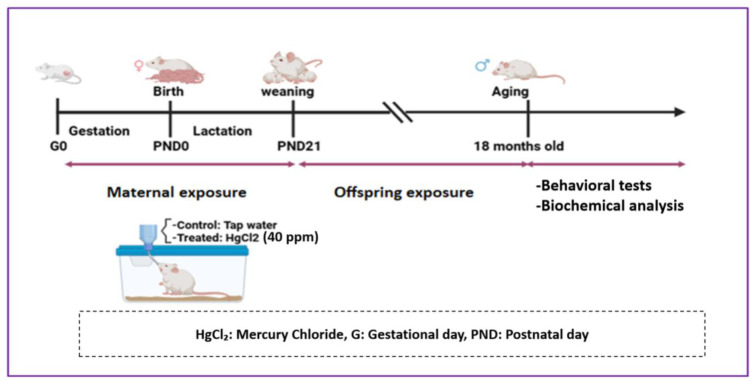
Experimental design and animal treatment.

At the beginning of the experiment, each virgin female was co-housed with a single male breeder, and the presence of a vaginal plug was checked daily in the early morning. Pregnancy was confirmed by the detection of a vaginal plug, designated as gestational day 0 (GD0). Pregnant females F0 were divided into two groups of 12 mice each: A control group receiving tap water and an HgCl_2_-treated group receiving freshly prepared drinking water containing 40 ppm of HgCl_2_. Solutions were provided in standard transparent drinking bottles under normal laboratory lighting. Although the stability of HgCl_2_ in water was not monitored, fresh solutions were prepared daily to ensure consistent exposure. Based on the literature [31], the photochemical transformation of Hg²⁺ under these conditions is negligible, and the solution was not expected to be affected by light. In adult male mice (3 months old), water consumption was monitored individually, with an average daily intake of 7.73 ml and an average body weight of 30 g, resulting in an estimated mercury dose of approximately 10 mg/kg/day. Each dam gave birth to 6–8 pups, with approximately half being male (3–4 pups per dam). F1 offspring from both groups remained with their mothers until weaning. After weaning, only male pups were used for the experiment, as we focused on male aging, and female pups were used for other experiments. The male F1 treated group continued to receive HgCl_2_ exposure until reaching 18 months of age (Figure 1). At this age, eight males were selected for experimental procedures, each originating from a distinct litter to control for potential litter effects. All animal experiments were conducted in accordance with approved institutional protocols and in compliance with the guidelines for the care and use of laboratory animals, as outlined in the European Council Directive 86/609/EEC on the protection of animals used for scientific purposes. The study was approved by the Council Committee of Research Laboratories at the Faculty of Sciences, Sultan Moulay Slimane University, Beni Mellal.

### 2.2. Behavioral tests

At the end of the mercury exposure, aged male mice (18 months) were subjected to behavioral testing from the least to most stressful ones in the following order: Open Field, Novel Object Recognition, and Y-maze. All mice were acclimated to the testing room for 30 minutes before each behavioral test, and testing occurred between 9:00 AM and 1:00 PM (first half of the light cycle). The apparatus was cleaned with a 10% ethanol solution between tests to eliminate any potential bias from olfactory cues.

#### 2.2.1. Open field test

The open field test (OF) was used to assess general locomotor activity level. The testing apparatus consisted of a square black Plexiglas arena (50 cm × 50 cm × 50 cm) with the floor divided into 25 equal squares for behavioral tracking. Individual mice were placed at the center of the open field arena and allowed to explore freely for a standardized 5-minute test session using a video camera located above the OF and monitored in another room via a closed-circuit TV camera. The levels of general activity were measured with two parameters, the total distance traveled, and average speed. Data were analyzed using ANY-Maze software (Stoelting Co., Wood Dale, IL, USA).

#### 2.2.2. Novel object recognition test

The Novel Object Recognition test (NOR) was conducted to evaluate recognition memory in rodents. The apparatus used in this test was a black Plexiglas open-field arena (50 cm × 50 cm × 50 cm). The procedure consisted of three sessions and was conducted over two days. Each animal explored the empty arena for 5 minutes during the habituation session (day 1). During the training session (day 2), the animals were exposed to the familiar arena with two identical objects (5 cm, neutral plastic, odorless, simple shape) placed at an equal distance of 10 cm away from the walls for 5 min. Two hours later, the test session began by allowing mice to explore the OF for 5 min in the presence of a familiar object and a novel object to test short-term recognition memory. Active investigation was considered when a mouse engaged an object with its nose pointed at it, no more than 2 cm away. Before each trial, the objects were carefully cleaned with a 30% ethanol solution and dried to eliminate any residual odor traces. The time spent exploring objects was recorded and the discrimination index was calculated [(time spent beside the novel object – time spent beside the familiar object) / total duration beside both objects] × 100. [32] Object positions (familiar vs. Novel) were counterbalanced across animals to prevent positional or object bias. During the training phase, no significant preference for either object was observed, confirming the absence of intrinsic bias. Data were analyzed using ANY-Maze software (Stoelting Co., Wood Dale, IL, USA).

#### 2.2.3. Ymaze test

The Y-maze spontaneous alternation test is based on the natural tendency of rodents to explore unfamiliar environments. Normal mice preferentially enter the arm they have not recently visited. In contrast, mice with impaired working memory show difficulty recalling their most recent location [33]. The Y-maze used in this study was made of Plexiglas and consisted of three identical arms (40 cm × 9 cm × 16 cm) arranged at 120° angles from one another. At the beginning of each trial, a mouse was placed at the end of one arm and allowed to explore freely for 5 minutes, while the experimenter remained out of sight. Sessions were video-recorded and analyzed using the ANY-maze tracking software (Stoelting Co., Wood Dale, IL, USA). An arm entry was defined according to the widely used “four-paw” criterion; an entry was counted when all four limbs of the animal were completely inside an arm [34]. Spontaneous alternation was defined as consecutive entries into all three arms, recorded as overlapping triplets (e.g., ABC and BCA). The percentage of these alternations was calculated to assess working memory performance.

### 2.3. Biochemical assays

#### 2.3.1. Sample collection

At the end of the treatment period, brains from control and treated mice (N = 7) were collected. All animals were euthanized using carbon dioxide (CO_2_). The hippocampus (right and left hemispheres) and the cerebellum were dissected on an ice-cold plate (4 °C), weighed, and stored at −20 °C until use.

#### 2.3.2. Tissue preparation

Hippocampus and cerebellum of the control and treated groups were homogenized in buffer solution TBS (50 mM Tris, 150 mM NaCl, pH 7.4), and then centrifuged at 10,000 × g for 15 min at 4 °C with centrifuge 5804 R (Eppendorf, Freshwater Blvd, Enfield, USA). Supernatants were collected and used to determine AChE Activity, the Malondialdehyde marker of lipid peroxidation, and the catalase and superoxide dismutase antioxidant enzymatic markers in the cell.

#### 2.3.3. Protein measurement

The Lowry method was used to assay the protein concentration [35] with bovine serum albumin as the standard.

#### 2.3.4. Acetylcholinesterase activity

The AChE specific activity was measured according to the method of Ellman [36] using acetylthiocholine iodide (Sigma-Aldrich, USA) as a substrate. The reaction mixture contained phosphate buffer (0.1 M, pH 8.0), acetylthiocholine iodide (0.075 M), and 5,5′-dithiobis-2-nitrobenzoic acid (DTNB; 0.01 M) (Sigma-Aldrich, USA). After the addition of brain structure, tissue homogenate (hippocampus and cerebellum) (30 min at room temperature), the hydrolysis rate of acetylcholine iodide was measured by a spectrophotometer (Selecta, Barcelona, Spain) at 412 nm. The enzyme activity was expressed as µmol AChE hydrolyzed/min/mg of protein.

#### 2.3.5. Malondialdehyde

The MDA levels in the hippocampus and cerebellum were measured according to the method of Buege and Aust [37]. A total of 125 µl of supernatant was homogenized by sonication with 50 µl of PBS and 125 µl of 20% TCA + 1% BHT (TCA-BHT) to precipitate proteins, and centrifuged (1000 × g, 10 min, 4 °C). Thereafter, 200 µl of supernatant was mixed with 40 µl of HCl (0.6 M) and 160 µl of TBA dissolved in Tris (120 mM). The mixture was heated at 80 °C for 10 min, and the absorbance was measured at 530 nm. The amount of thiobarbituric acid reactive substances (TBARS) was calculated using a molar extinction coefficient of 1.56 × 105 M/cm.

#### 2.3.6. Catalase (CAT)

The CAT activity was measured at 240 nm using a UV/visible spectrophotometer by the variation of the optical density consecutive to the disproportionation of hydrogen peroxide (H_2_O_2_) [38]. For the enzyme reaction, 20 µl of supernatant was added to 780 µl of phosphate buffer saline (PBS) (0.1 M, pH 7.4) and 200 µl of H_2_O_2_ (0.5 M).

#### 2.3.7. Superoxide dismutase (SOD)

The SOD activity was measured by the method of Asada [39]; 0.05 ml of the supernatant was added to 0.1 ml of a mixture containing methionine (13 mM) and Na2 EDTA (0.1 mM), 0.8922 ml of phosphate buffer (50 mM, pH = 7.8), 0.95 ml of phosphate buffer, 0.088 ml of NBT (2.64 mM), and 0.0226 ml of riboflavin (0.26 mM).The reduction of NBT was estimated after 20 min at a wavelength of 580 nm against white.

### 2.4. Data analysis and statistics

Statistical analysis and figure generation were performed using Graphpad Prism 9.0. Data normality and homogeneity of variances were systematically verified before parametric testing. Following confirmation of these assumptions (p > 0.05 for normality and variance equality tests), Student's unpaired t-test was applied for group comparisons. Results are presented as mean ± SEM, with p < 0.05 considered statistically significant.

## 3. Results

### 3.1. Behavioral tests

#### 3.1.1. Locomotor activity

**Figure 2.**
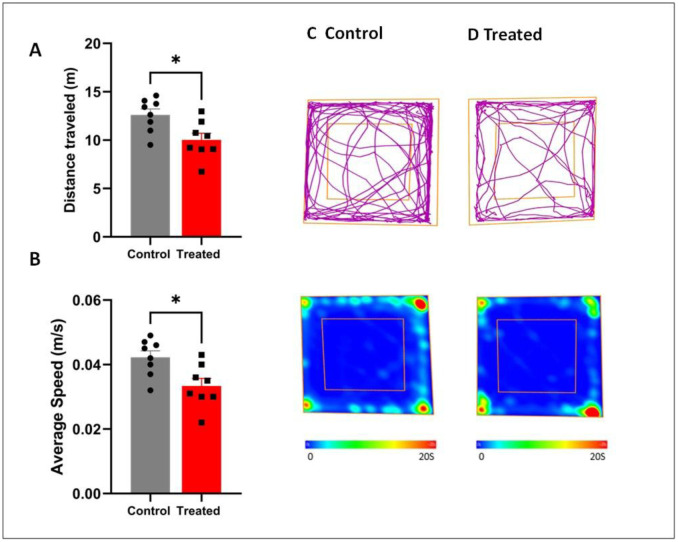
Effect of HgCl_2_ on locomotor activity in the OF test. A: Total distance traveled. B: Average speed. C: Trajectories and heat plots mapping the occupied areas of control mice. D: Trajectories and heat plots mapping the occupied areas of treated mice. Each value represents the mean ± S.E.M. Student's t-test: *p < 0.05 compared to the control group (n = 8/group).

The results in the open field test showed that male aged mice exposed to HgCl_2_ from gestation to old age exhibited a significant decrease in locomotor activity compared to the control aged mice, as indicated by their trajectories and heat plot mapping of the occupied areas in the OF test (Figure 2). Student t-test analysis revealed that treated mice displayed a significantly lower total traveled distance (p = 0.0135; t = 2.824; df = 14) and average speed (p = 0.0121; t = 2.881; df = 14) compared to the control (Figure 2).

#### 3.1.2. Learning and memory

**Figure 3.**
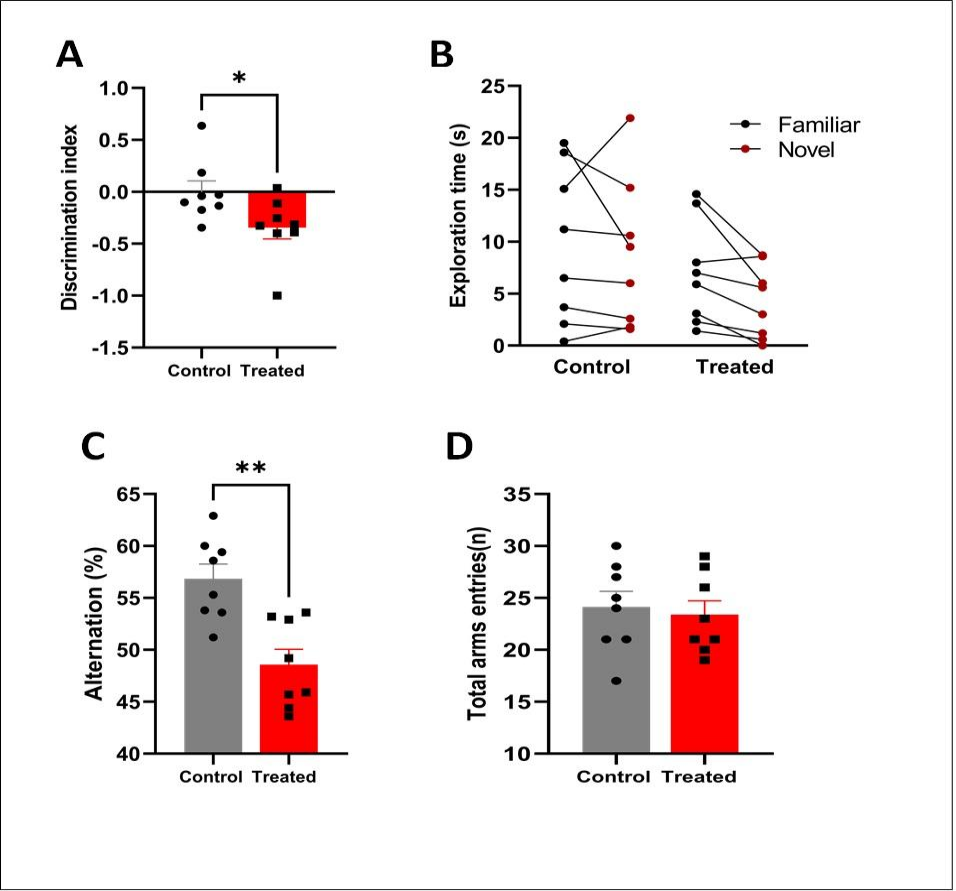
Effect of HgCl_2_ on memory function. A: Recognition memory and B: Exploration time evaluated by the object recognition test. C: Spatial working memory and D: Total arm entries evaluated by the Y-maze test. Results are expressed as mean ± SEM. Significant effects were revealed by Student's t-test: *p < 0.05; **p < 0.01; compared to the control group (n = 8/group).

*Novel Object Recognition test:* The Novel Object Recognition test showed that chronic exposure to HgCl_2_ until 18 months impaired memory recognition in aged mice compared to controls, as shown in Figure 3, with a significant decrease in the discrimination index (p = 0.0377; t = 2.295; df = 14).

*Y-maze test:* In this test, we assessed the impact of HgCl_2_ on the short-term spatial working memory of mice in the Y-maze test. Our results showed that the percentage of alternation (p = 0.0012; t = 4.058; df = 14) in the three arms was significantly lower after chronic exposure to mercury chloride compared to the controls (Figure 3).

### 3.2. Biochemical assays

#### 3.2.1. Lipid peroxidation activity

**Figure 4.**
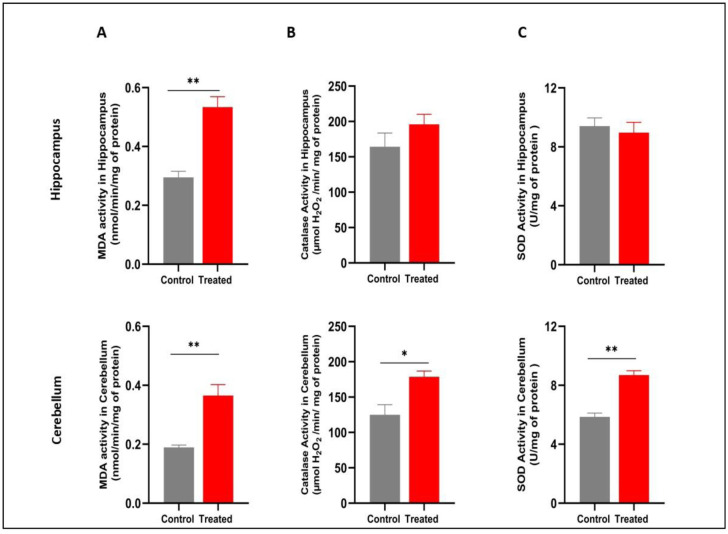
Effect of HgCl_2_ treatment on oxidative stress in the cerebellum and hippocampus. A: MDA activity. B: CAT activity. C: SOD activity. Results are presented as mean ± SEM. Student's t-test: *p < 0.05, **p < 0.01; compared to the control group (n = 7/group).

To assess lipid peroxidation in the hippocampus and cerebellum, the MDA level was measured. The results revealed that chronic exposure to HgCl_2_ significantly increased this parameter in the hippocampus (p = 0.0043; t = 5.822; df = 4), with a similar effect observed in the cerebellum of treated mice compared to the control group (p = 0.0095; t = 4.671; df = 4) (Figure 4).

#### 3.2.2. Antioxidant enzyme activities

Data analysis revealed that HgCl_2_ exposure significantly increased catalase activity in the cerebellum (p = 0.0302; t = 3.292; df = 4), but no significant difference was observed between treated and control aged mice in the hippocampus (p = 0.2595; t = 1.313; df = 4). The SOD activity results showed that mercury chloride induced a significant increase in the cerebellum (p = 0.0018; t = 7.414; df = 4), whereas no significant difference was found in the hippocampus between the treated and control groups (p = 0.6478; t = 0.4931; df = 4) (Figure 4).

#### 3.2.3. Acetylcholinesterase activity

**Figure 5.**
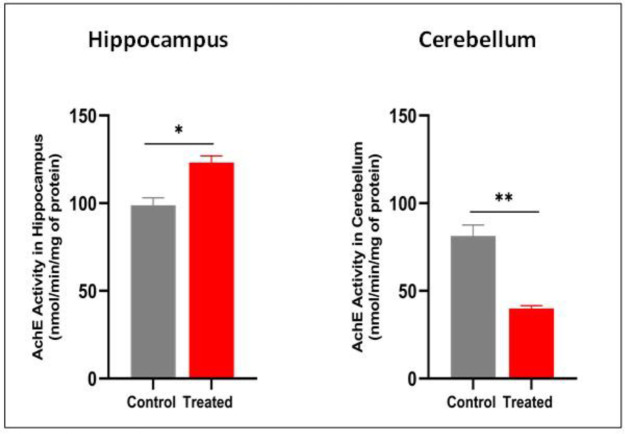
Effect of HgCl_2_ treatment on AChE activity in cerebellum and hippocampus. A: AChE in Cerebellum. B: AChE in Hippocampus. Results are presented as mean ± SEM. Student's t-test: *p < 0.05, **p < 0.01; compared to the control group (n = 7/group).

As shown in the results, the exposure to HgCl₂ significantly inhibited AChE activity in the cerebellum of treated mice compared to the controls (p = 0.003; t = 6.429; df = 4). In contrast, AChE activity was significantly increased in the hippocampus of treated animals following HgCl_2_ exposure (p = 0.0128; t = 4.282; df = 4) in these mice (Figure 5).

## 4. Discussion

Mercury is an environmental factor that may contribute to accelerated aging due to its genotoxic, autoimmune, inflammatory, and pro-oxidant effects [40],[41], which promote many disorders related to aging [42],[43].We aim to investigate how chronic HgCl_2_ exposure may accelerate the onset of neurological disorders associated with aging. The results indicated that this intoxication induces accelerated neurological alterations that characterize aging, including a decline in memory abilities and impaired locomotor activity. These behavioral deficits were accompanied by biochemical alterations in brain structures involved in the regulation of these functions, likely resulting from mercury accumulation in the brain of treated mice following prolonged exposure.

In the first part of our study, we assessed that chronic exposure to mercuric chloride until aging induced a significant impairment in recognition memory, where the treated groups exhibited a lower discrimination index compared to their control, as shown in the recognition object task. We also observed a low alternation index in treated animals compared to the controls in short-term spatial working memory. These behavioral alterations may be linked to cerebral disturbances, particularly in the hippocampus, which has been shown to play a crucial role, especially in memory and learning. Moreover, we found that chronic exposure to mercury chloride increased the level of lipid peroxidation (MDA) in the hippocampus without a concomitant induction of major antioxidant enzymes (SOD, CAT), indicating an insufficient adaptive response. This limitation may be explained by the intrinsic characteristics of hippocampal neurons: High energy demand, intense mitochondrial activity, and continuous production of ROS/RNS, which make them particularly vulnerable to oxidative damage [14],[44]. Moreover, pyramidal neurons in the CA1 and CA3 regions, as well as granule cells in the dentate gyrus, have lower basal antioxidant defenses than other neuronal populations, reducing their capacity to neutralize ROS accumulation [45],[46]. In our previous study in adult mice [24], we reported the same result, as did several other studies on adult rats. Aragão et al. [47] and Behzadfar et al. [48] have also reported hippocampal dysfunction associated with oxidative stress, tissue damage, and alterations in mitochondrial function in adult animals after a chronic exposure to mercury chloride.

Alternatively, this memory impairment could be linked to AChE alteration in the hippocampus, which is a key enzyme in regulating acetylcholine levels, a neuromodulator that plays a central role in various brain functions, particularly hippocampus-dependent memory [49],[50]. Our results showed a significant increase in AChE activity in the hippocampus of aged mice exposed to HgCl_2_, compared to controls. This finding was not observed in adult mice in our previous study, and it may be attributed to the prolonged duration of exposure and increased mercury accumulation in the hippocampus with aging.

The alteration of this activity may be directly related to high levels of ROS in this structure: As mentioned, the hippocampus is known for its intrinsically high production of reactive oxygen species (ROS) [14],[46], which is exacerbated by the presence of mercury [47], and its inadequate antioxidant response. Furthermore, elevated ROS levels in the brain region may interfere with the cholinergic system and disrupt its function. Although comparable data for mercury exposure remain limited, researchers using other agents have demonstrated that oxidative stress can induce an increase in AChE activity, notably in retinal cells exposed to amyloid-β peptide [51]. Furthermore, inorganic mercury (HgCl_2_) is shown to directly inhibit cholinesterase activity through interactions with sulfhydryl (thiol) groups on the enzyme, as demonstrated in vitro and in vivo [30],[52]. Therefore, the increase in enzymatic activity observed in this study may reflect a compensatory or maladaptive mechanism of the cholinergic system in response to mercury-induced AChE inhibition, assuggested by Moretto et al. [53]. Despite the exact molecular mechanisms remaining unclear, the cholinergic system appears to be a major target of mercury neurotoxicity [53].

In the second part of this work, we observed a significant reduction in locomotor activity in the OF test in aged mice chronically exposed to HgCl_2_, characterized by decreased speed and distance traveled compared to the control mice. These findings could be partially explained by the increase in oxidative stress, as evidenced by elevated lipid peroxidation (MDA) levels and increased activity of primary antioxidant enzymes, such as SOD and catalase, in the cerebellum that are involved in movement and coordination. This dysregulation of oxidative status resulting from excessive oxidant production or impaired antioxidant defenses is widely recognized as a key consequence of heavy metal toxicity in animals and humans [54]. The presence of mercury in the cerebellum may induce excessive oxidative stress and overproduction of ROS, as reported in studies describing cadmium and mercury-induced oxidative, neurobehavioral, and histological alterations in the cerebellum [55]. Excessive ROS can impair mitochondrial function in the brain region by inducing mitochondrial DNA mutation, damaging components of the mitochondrial respiratory chain altering mitochondrial membrane permeability, and disrupting Ca²⁺ homeostasis and mitochondrial defense systems [56]. Similar findings were reported by Bellum et al. [57], who showed that moderate exposure to methylmercury in aged mice (16–20 months) led to reduced locomotor activity, increased production of ROS, and decreased mitochondrial membrane potential in cerebellar granule cells [58]. Other researchers assessed an antioxidant enzyme imbalance and histopathological changes in the cerebellum after intoxication of adult rats over 14 days with HgCl₂ and cadmium; these changes included Purkinje cell loss and disorganization of cerebellar layers, indicating a neurodegenerative process [56]. Moreover, mercury has been shown to directly inhibit mitochondrial respiratory chain complexes I-IV through interactions with sulfhydryl (-SH) groups and iron-sulfur centers, thereby disrupting electron transfer and cerebral oxidative phosphorylation, as reported in rat cells [59]. Such mitochondrial dysfunction may also impair synaptic transmission within cerebellar circuits by limiting ATP availability and disrupting Ca^2+^-dependent neurotransmitter release, compromising neuronal firing and motor coordination. These mitochondrial and synaptic alterations provide a mechanistic basis for the reduced locomotor activity observed in mercury-treated mice.

Despite these alterations, the cerebellum appears to exhibit a higher regional antioxidant capacity, characterized by greater basal and inducible antioxidant enzyme activity than the hippocampus. Moreover, studies on aged rats have shown that the cerebellum displays significantly higher superoxide dismutase activity than other brain regions, whereas glutathione peroxidase activity declines in the hippocampus during aging, highlighting regional differences in antioxidant defenses [60]. Despite this enhanced antioxidant capacity, excessive ROS production in the cerebellum following mercury exposure may overwhelm these protective mechanisms, leading to mitochondrial dysfunction, impaired synaptic transmission, and locomotor deficits.

In parallel with the oxidative stress finding, we observed a significant inhibition of AChE activity in the cerebellum of aged mice exposed to HgCl_2_, which may represent a mechanism underlying the observed alterations in locomotor activity.

In our work on adults, we also assessed decreased AChE activity in this region, although without detecting any locomotor deficit, suggesting that motor impairments may appear only after prolonged exposure, likely due to progressive mercury accumulation in cerebellar tissues. AChE inhibition could thus contribute to cholinergic neurotransmission dysregulation, leading to reduced motor activity. This inhibition may be explained, in part, by direct inhibition through binding to the enzymes, as demonstrated in vivo and in vitro [30],[52]. Moreover, this interaction could induce conformational changes in AChE and reduce its catalytic efficiency.

The decrease in AChE activity observed in the cerebellum may also be linked, as discussed for the hippocampus, to the pronounced increase in lipid peroxidation resulting from mercury accumulation in this structure [61]. Contradictory findings have been reported, showing increased ROS levels accompanied by elevated AChE activity in the cerebellum following mercury chloride exposure [62]. Such discrepancies may be attributed to differences in dose, exposure duration, or species, as observed in rat studies in which cerebral and cerebellar oxidative stress correlated with increased AChE activity. At the cerebellar level, although ROS are also produced, antioxidant mechanisms appear more effective in limiting their accumulation. The inhibition of AChE in this brain region likely reflects the direct deleterious effects of ROS rather than compensatory upregulation. The absence of a compensatory increase in AChE activity in the cerebellum may indicate a more stable or less plastic cholinergic regulation, with distinct transcriptional and post-transcriptional control mechanisms compared to the hippocampus.

Taken together, it appears that the intoxication procedure, which began during gestation and continued into advanced age, enabled us to highlight the impact of early and continuous exposure to HgCl_2_ on the hippocampus and cerebellum. This model incorporates in utero exposure via maternal intoxication, a period of high developmental vulnerability, during which even low doses can induce silent alterations that are not immediately detectable but may manifest later when the individual's functional capacity is challenged. Aging can be considered such a condition, as is associated with a decline in physiological functions and compensatory reserves [58],[63].

The alterations observed in the hippocampus and cerebellum may therefore result from this early and prolonged exposure. These periods correspond to critical windows of brain development, including neurogenesis, neuronal migration, synaptogenesis, and circuit formation, all of which are essential for the proper functioning of these two structures [64],[65].

Furthermore, studies on rats have shown that prenatal exposure to HgCl_2_ leads to higher mercury accumulation in the hippocampus than in the cerebellum of offspring and infants [66], which may explain the regional differences observed in our study at the biochemical and functional levels. These differences do not necessarily reflect distinct initial sensitivities but may emerge during aging when defense systems become compromised, thus explaining variations in antioxidant response and AChE activity modulation. This regional divergence is also consistent with the observation of Cace et al. [67], who reported that prenatal mercury exposure, even at low doses, reduces cerebellar size in neonates, suggesting that the cerebellum remains vulnerable during development.

These early insults, combined with differential mercury accumulation, render the hippocampus and cerebellum particularly susceptible to oxidative stress and cholinergic dysregulation in advanced age, contributing to memory deficits observed in our study. This conceptual framework aligns with the idea that developmental neurotoxicity can program long-lasting alterations in cognitive function [64]. Finally, studies by Stern et al. [68] suggest that early and continuous exposure results in long-term mercury accumulation in the brain, with prolonged neurotoxic effects.

## 5. Conclusions

Our results indicate that exposure to HgCl_2_ from gestation through aging accelerates the aging process, particularly affecting two key functions often impaired with age: Memory and locomotor activity, as well as, more broadly, neurodegenerative processes. The observed alterations in brain functions may result from the accumulation of oxidative damage and disruptions in AChE activity. Furthermore, our data reveal region-specific responses in the brain: The hippocampus and cerebellum exhibit distinct profiles of oxidative stress and cholinergic activity, suggesting that these structures respond differently to the neurotoxic effects of mercury. However, certain limitations remain, namely the lack of precise measurements of water consumption, the exact amount of mercury ingested, and the quantification of mercury and acetylcholine levels in the hippocampus and cerebellum. Despite these limiations, our findings provide valuable insights into the region-specific effects of mercury on oxidative stress and cholinergic activity. These results underscore the importance of this study for a better understanding of regional mercury neurotoxicity during aging and provide a framework for guiding future research.

## Use of AI tools declaration

The authors declare they have not used Artificial Intelligence (AI) tools in the creation of this article.
